# Implantable Bioresorbable Scaffold with Fucosylated Chondroitin Sulfate as a Promising Device for Delayed Stimulation of Hematopoiesis

**DOI:** 10.3390/md23090344

**Published:** 2025-08-28

**Authors:** Natalia Y. Anisimova, Olga V. Rybalchenko, Natalia S. Martynenko, Georgy V. Rybalchenko, Elena A. Lukyanova, Maria I. Bilan, Anatolii I. Usov, Mikhail V. Kiselevskiy, Nikolay E. Nifantiev

**Affiliations:** 1 N.N. Blokhin National Medical Research Center of Oncology (N.N. Blokhin NMRCO) of the Ministry of Health of the Russian Federation, 115478 Moscow, Russia; n_anisimova@list.ru (N.Y.A.); kisele@inbox.ru (M.V.K.); 2 A.A. Baikov Institute of Metallurgy and Materials Science of the Russian Academy of Sciences, 119334 Moscow, Russia; rybalch@mail.ru (O.V.R.); nataliasmartynenko@gmail.com (N.S.M.); helenelukyanova@gmail.com (E.A.L.); 3 P.N. Lebedev Physical Institute of the Russian Academy of Sciences, 119991 Moscow, Russia; rybalchenkogv@lebedev.ru; 4 N. D. Zelinsky Institute of Organic Chemistry of the Russian Academy of Sciences, 119991 Moscow, Russia; bilan@ioc.ac.ru (M.I.B.); usov@ioc.ac.ru (A.I.U.)

**Keywords:** fucosylated chondroitin sulfate, hemo-stimulation, implant, scaffold

## Abstract

The aim of this study was to evaluate the prospects of using natural fucosylated chondroitin sulfate (FCS) from the sea cucumber *Cucumaria japonica* as the active component of an implantable biodegradable scaffold to stimulate hematopoiesis in mice with cyclophosphamide (CPh)-induced myelosuppression. The scaffolds were based on bioresorbable Fe–Mn–C and Fe–Mn–Pd alloys after equal-channel angular pressing (ECAP). The efficiency of the developed constructs with FCS was compared with the activity of the same scaffolds loaded with recombinant human granulocyte colony stimulating factor, as well as solutions of these active compounds administered subcutaneously after the end of the cyclophosphamide (CPh) course. It was found that implantation of the Fe–Mn–C scaffold loaded with FCS most effectively stimulated hematopoiesis, providing a complex effect. This design of the developed constructs contributed to an increase in the concentration not only of leukocytes and neutrophils, but also platelets in the blood, promoted the proliferation of bone marrow cells, increasing the concentration of Ki-67(+) cells, and contributed to the restoration of the morphology of the animals’ spleen.

## 1. Introduction

It is well known that neutropenia is one of the most common complications of chemotherapy in cancer patients, leading to an increased risk of infection [1]. Recombinant human granulocyte colony stimulating factor (rG-CSF; filgrastim) is widely used to reduce the severity and duration of neutropenia [2]. After a single injection of filgrastim (5 μg/kg), an increase in white blood cells (WBCs) in the blood is observed after 12–14 h [3], and the average duration of neutropenia, according to [4], is 3 days. Granulocyte colony stimulating factor (G-CSF) stimulates the survival and proliferation of neutrophil precursors and the function of mature neutrophils, increases the production of a whole spectrum of cytokines IL-10, IL-8, IL-6, and IL-1 and soluble tumor necrosis factor receptors (sTNF-Rs), reduces the concentrations of IFN-λ, TNF-α, as well as granulocyte-macrophage colony-stimulating factor (GM-CSF), and therefore can play a significant role in the development of a cytokine storm [5,6].

Filgrastim is characterized by a short half-life (2–4 h) with elimination from the body by renal clearance and G-CSF receptor-associated uptake by neutrophils [7]. Lenograstim, which is a glycosylated recombinant human G-CSF similar in pharmacokinetics to filgrastim, is also used in clinical practice. These drugs are recommended for use no earlier than 24 h after a course of chemotherapy [8]. After subcutaneous and intravenous administration, filgrastim and lenograstim are eliminated from the body quite quickly. Therefore, in practical medicine, the use of filgrastim and lenograstim is usually provided as a daily course to achieve an adequate increase in neutrophil concentration, which requires a different number of administrations, usually 5 or more [9,10].

In practice, compliance with these instructions presents, in some cases, significant difficulties, since it requires the patient to re-visit a medical institution and receive additional injections. The problem is that exactly during this period the maximum manifestation of systemic chemotherapy side effects (vomiting, diarrhea, fever, neurotoxicity, etc.) is observed. In this regard, some patients do not receive this maintenance therapy partially or at all, which negatively affects the overall outcome of treatment.

In order to reduce the effective course of G-CSF, various approaches are being developed aimed at prolonging the circulation of the active substance in the body. This can be achieved by placing the active compound on a biodegradable platform or in a scaffold. In this case their kinetics of bio-resorption will determine the bioavailability of the drug. Additionally, creating conjugates can change the pharmacokinetics of the active compound. Thus, in recent years, the drug pegfilgrastim, of prolonged action, containing pegylated filgrastim, has been actively used in clinics. The median serum half-life of pegfilgrastim is 42 h due to the impossibility of clearance by the kidneys due to the large size of the conjugate molecule. As a result, it can be administered to patients once, resulting in the effect of neutropenia stimulation corresponding to 11 injections of filgrastim [11,12]. However, pegfilgrastim cannot be administered earlier than one day after the end of chemotherapy, and its therapeutic effect is only realized in patients with neutropenia.

Meanwhile, intensive chemotherapy can cause myelosuppression, which is manifested in a decrease in the content not only of white blood cells (WBCs), but also of red blood cells (RBCs) and platelets (PLT) in the circulating blood. In this case, along with a violation of anti-infective immunity due to leukopenia, patients experience anemia, leading to fatigue, and thrombocytopenia, and thus to fear and anxiety [13]. These symptoms not only reduce quality of life, but can cause the patient’s death. Particularly dangerous is pancytopenia, when a simultaneous decrease in the content of WBCs, RBCs and PLT in the patient’s blood is noted.

Myelosuppression is often the cause of low efficiency of cancer treatment due to increase of intervals between chemotherapy courses, cancellation of effective cytostatics, or reduction of their dose. Obviously, such patients require supportive therapy to restore the cellular composition of the blood, including not only G-CSF containing drugs, but also transfusion of erythropoiesis-stimulating agents, blood or platelet concentrate [14,15]. However, erythropoiesis-stimulating agents are not always effective and can induce thromboembolism [16], while the therapeutic effect of blood or platelet transfusion is short-lived and can be accompanied by complications.

Therefore, the urgent task is not only the development of new medicinal forms of hemo-stimulants, but also the search for new active compounds that are promising for the prevention of myelosuppression due to the effect on various blood cells. In this regard, of particular interest are studies of promising candidates among sulfated oligo- and polysaccharides of natural origin, which include fucoidans [17], xylofucan [18], fucosylated chondroitin sulfate (FCS) and other hexosaminoglycans [19,20,21,22,23,24]. In the course of previous studies, it was shown that individual representatives of this class, extracted from brown algae and sea cucumbers, have a pronounced ability to influence hematopoietic cells and restore hematopoiesis in experimental animals with cyclophosphamide (CPh)-induced myelosuppression [19,20,21]. The studies conducted in [22,23] made it possible to identify a number of promising representatives of the FCS class, as well as to determine an effective therapeutic dose that made it possible to achieve an effect similar to that of filgrastim. It was shown that parenteral administration of solutions of these FCS contributed to a reliable increase in the number of neutrophils, leukocytes, and erythrocytes in the blood, along with stimulation of the proliferation of hematopoietic cells of the bone marrow ex vivo and restoration of the functions of circulating immunocompetent cells in comparison with the control, where the animals recovered after the administration of CPh without additional therapy.

The aim of this study was both to confirm the prospects of using natural FCS extracted from the sea cucumber *C. japonica* to stimulate hematopoiesis against the background of CPh-induced myelosuppression, and to evaluate the effectiveness of using this compound as part of an implantable structure based on a biodegradable scaffold to implement a delayed therapeutic effect. Biodegradable Fe–Mn-based alloys, which are considered promising for the manufacture of both intravascular stents and implants for osteosynthesis [25,26,27,28], were chosen as the material for the scaffold shell. They combine the ability to be biodegraded with low toxicity and acceptable mechanical properties that determine the functionality of the scaffold. The specific strength of such alloys along with sufficient plasticity allows for the production of a thin-walled structure that protects the loaded active compound from contact with tissues during subcutaneous administration and prevents its rapid degradation after implantation. Since the corrosion rate of Fe–Mn-based alloys is quite low [25,26,27,28], a combined effect of additional alloying and severe plastic deformation by equal-channel angular pressing (ECAP) was used to increase it [29,30].

## 2. Results

### 2.1. Material Characterization

After severe plastic deformation by ECAP, the Fe–Mn–C and Fe–Mn–Pd alloys had an ultrafine-grained austenitic structure. The size of the structural elements was 100–250 nm, with release of α-Mn nanoparticles in the Fe–Mn–C alloy [30] and Mn_2_Pd_3_ intermetallic in the Fe–Mn–Pd alloy [29]. The structure formed by ECAP led to an increase in the strength characteristics and corrosion rate of the alloys used (Table 1).

### 2.2. Characteristics of Alloy Degradation Products

**Figure 1:**
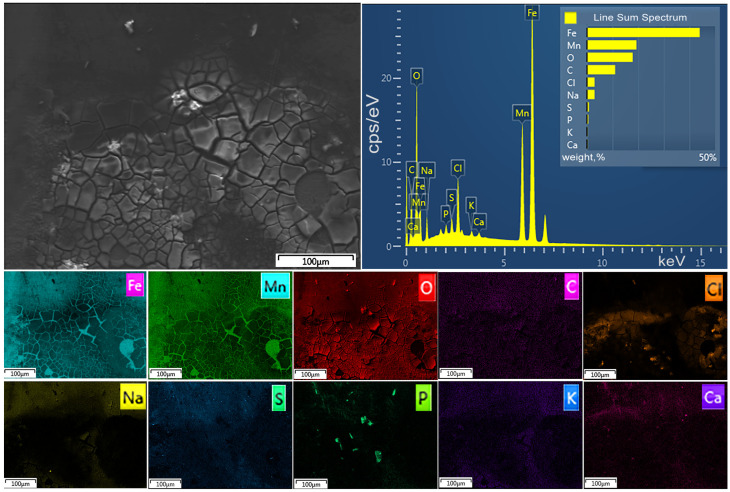
SEM images of the surface of ECAP-treated Fe-27Mn-0.2C alloy after incubation in RPMI-1640 for 7 days.

**Figure 2:**
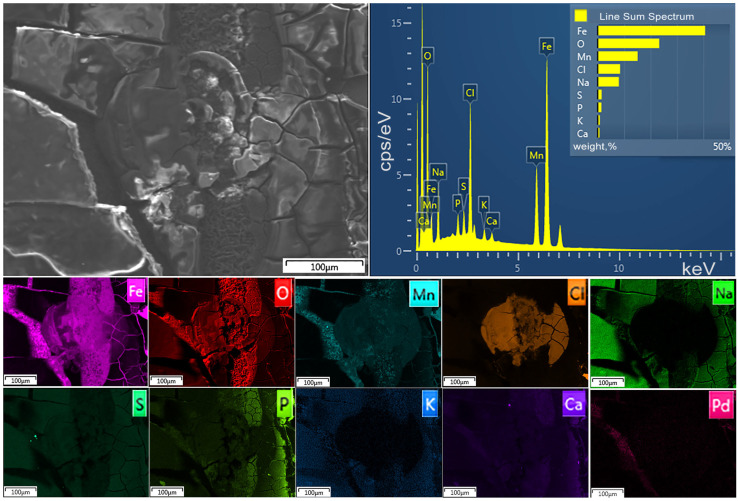
SEM images of the surface of ECAP-treated Fe-22Mn-0.9Pd alloy after incubation in RPMI-1640 for 7 days.

Figure 1 and Figure 2 show SEM images of the corroded surface of Fe–Mn–C (Figure 1) and Fe–Mn–Pd (Figure 2) alloy samples treated by ECAP after their incubation in the culture medium for 7 days. A fairly thick layer of corrosion products was observed on the surface of the alloys. The corroded surface after incubation of the samples was a cracked layer, where the corrosion products were quite firmly attached to the alloy but were already starting to peel off. At the same time, the composition of the corrosion product layer of the two alloys is almost identical and corresponds to that shown in the elemental mapping, differing in a small Pd content.

Based on the presence of elements on the surface of the samples in the composition of the corrosion products, one can assume a mixture of oxides, hydroxides and phosphates on the surface of the samples.

Typically, culture media contain Ca^2+^, Mg^2+^, Na^+^, K^+^, Cl^−^, SO_4_^2–^, PO_4_^3−^ and HCO_3_^−^ ions [31]. Most of them are represented in the elemental mapping of alloys after degradation in the culture medium of Fe–Mn–C (Figure 1) and Fe–Mn–Pd (Figure 2).

### 2.3. Administration of Active Compounds to Animals

**Figure 3:**
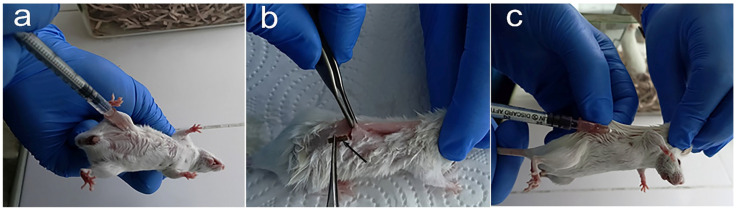
Intraperitoneal injection of CPh in mouse (**a**) followed by administration of hemo-stimulator by implantation of the loaded scaffold (**b**) or subcutaneously (**c**). The arrow indicates the device for delayed hemo-stimulation before the subcutaneous implantation.

The Fe–Mn-C and Fe–Mn-Pd alloy scaffolds loaded with FCS or rG-CSF were implanted subcutaneously in mice with induced immunosuppression immediately after the end of the last course of CPh (Figure 3). In order to evaluate the effect of the developed devices over prolonged action, mice from other groups were treated with FCS or rG-CSF subcutaneously, simultaneously with Matrigel.

### 2.4. Hematological Parameters

**Figure 4:**
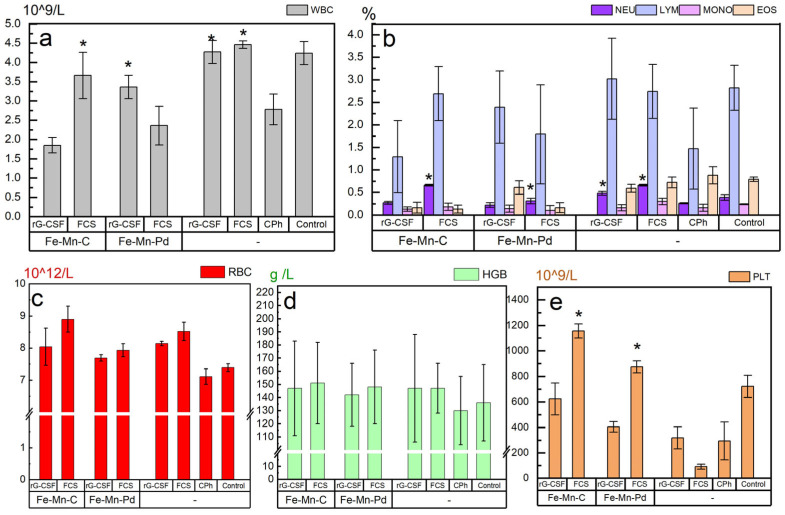
The effect of FCS and rG-CSF, administered subcutaneously in Fe–Mn–C and Fe–Mn–Pd alloy scaffolds or in solution form to mice with CPh-induced immunosuppression, on the recovery of leukocyte (**a**), neutrophil, lymphocyte, monocyte and eosinophil (**b**), erythrocyte (**c**) hemoglobin (**d**) and platelet (**e**), * *p* < 0.05 (vs. CPh).

Analysis of hematological parameters of mice with immunosuppression without treatment (group CPh) shows that a course of the cytostatic drug induced a dramatic decrease in the concentration of hemoglobin, platelets and WBCs in the blood compared to the intact control (Figure 4). In this case, the content of neutrophils and lymphocytes decreased most significantly.

**Figure 5:**
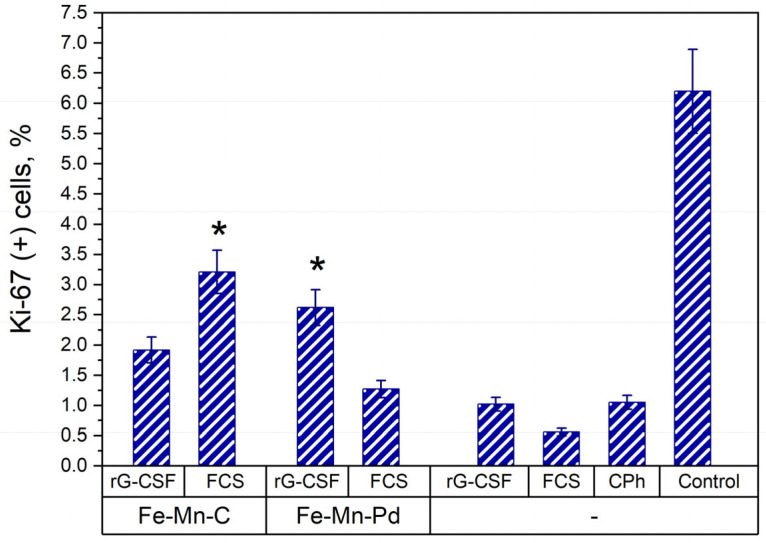
Concentration of Ki-67 labeled cells in the bone marrow of mice with CPh-induced immunosuppression treated by FCS and rG-CSF loaded into Fe–Mn–C and Fe–Mn–Pd alloy scaffolds or in solution form, in comparison with mice with CPh-induced immunosuppression without therapy and intact control, * *p* < 0.05 (vs. CPh).

At this stage of the research, our task was not to optimize the scaffold composition, but to identify the fundamental possibility of the use of the described scaffolds to manage a delayed effect of FCS and rG-CSF. As expected, the study revealed a number of desirable statistically significant results (Figure 4 and Figure 5, * *p* < 0.05).

The study compared the effect of FCS in the Fe–Mn–C+FCS, Fe–Mn–Pd+FCS and FCS groups, when administered subcutaneously to mice with CPh-induced immunosuppression. FCS administered in the form of a solution demonstrated the greatest effect on WBC recovery. This result was the best among all the studied groups. The most pronounced statistically significant restoration of WBCs under the influence of hemo-stimulants was observed in the Fe–Mn–C+FCS, FCS and rG-CSF groups: on average, the concentration of these cells was increased in comparison with the CPh group by 1.3, 1.6 and 1.7 times (*p* < 0.05), respectively (Figure 4a). In the Fe–Mn–Pd+rG-CSF group, a reliable WBC increase was also observed, but the effect was weaker: the number of cells increased by 1.2 times. It can also be stated that the treatment of animals with FCS led to an increase in the concentration of neutrophils in the blood. This is also the best result among all the groups considered. Both in the Fe–Mn–C+FCS group and in the FCS group, the neutrophil concentration increased by 2.7 times compared to the CPh group, exceeding even this indicator in the intact control group (Figure 4b). In the rG-CSF group, a 2-fold increase in neutrophil concentration was also observed compared to the CPh group. The concentrations of lymphocytes, monocytes, and eosinophils varied widely between the groups, with no evidence of significant differences between them.

The treatment of animals with FCS or rG-CSF, both using scaffold and injection solution, resulted in a tendency to increase the concentration of RBCs and hemoglobin without significant differences between groups (Figure 4c,d). The most active statistically significant restoration of platelet concentration was observed in the Fe–Mn–C+FCS and Fe–Mn–Pd+FCS groups (on average 3.7 and 2.8 times higher compared to the CPh group (*p* < 0.05), respectively) (Figure 4e). In the Fe–Mn–C+rG-CSF group, a tendency to increase the amount of PLT by 2 times was also noted compared to the CPh group.

### 2.5. Proliferative Activity of Cells

Ki-67 is a well-known marker of actively dividing cells. The number of cells labeled Ki-67 was used for assessing the proliferative activity of bone marrow cells after treating animals with scaffolds for delayed stimulation of hematopoiesis. The obtained data showed, as expected, that treatment of animals by CPh resulted in suppression of the number of proliferating cells in their bone marrow (Figure 5). In Figure 5, the results of the effect of preparations Fe–Mn–C+FCS and Fe–Mn–Pd+rG-CSF are statistically significant (* *p* < 0.05). These results demonstrate the best efficiency when Fe–Mn–C+FCS is used, which significantly increases the concentration of Ki-67 cells in the bone marrow of mice with immunosuppression. In the other groups, no reliable differences were noted compared to the CPh group (*p* > 0.05).

**Figure 6:**
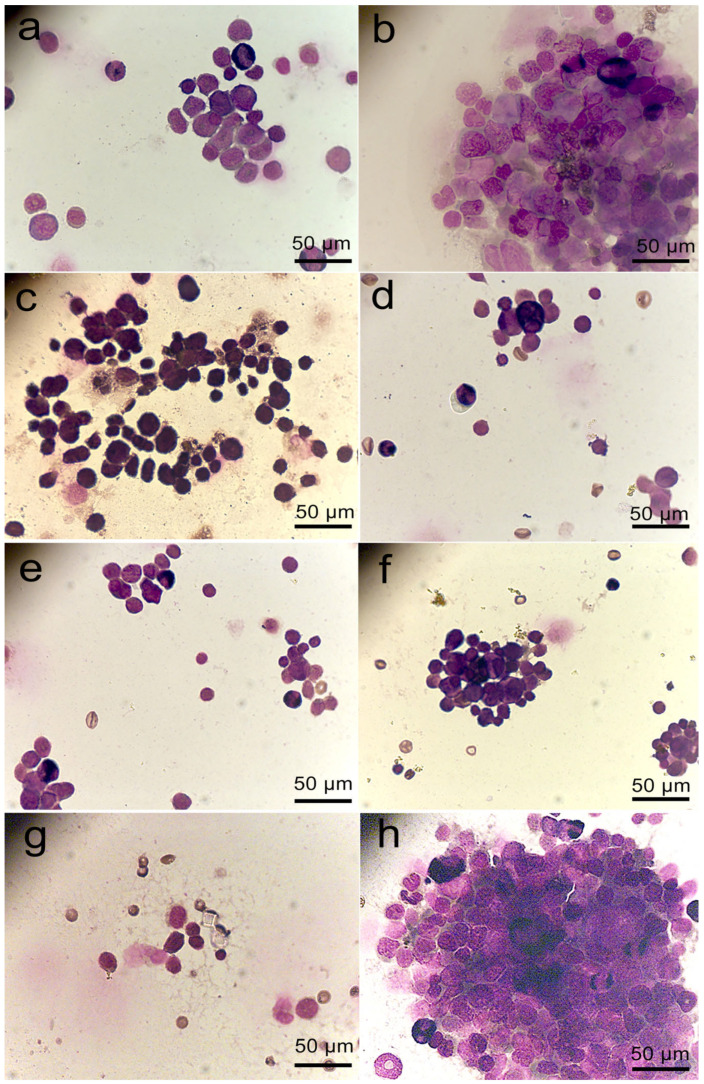
Proliferation of bone marrow cells in mice with CPh-induced immunosuppression after implantation of Fe–Mn–C+rG-CSF (**a**), Fe–Mn–C+FCS (**b**), Fe–Mn–Pd+rG-CSF (**c**), Fe–Mn–Pd+FCS (**d**) scaffolds or subcutaneous administration of rG-CSF (**e**) and FCS (**f**) solutions, in comparison with mice with CPh-induced immunosuppression without therapy (**g**) and intact control (**h**).

The data on the concentration of Ki-67(+) cells in general were confirmed by the results after assessing the morphology of bone marrow cells. The largest number of pools of dividing cells in the bone marrow was observed in animals of the Fe–Mn–C+FCS group (Figure 6b). In the other groups, the restoration of the bone marrow proliferative activity was slower.

### 2.6. Spleen Morphology

**Figure 7:**
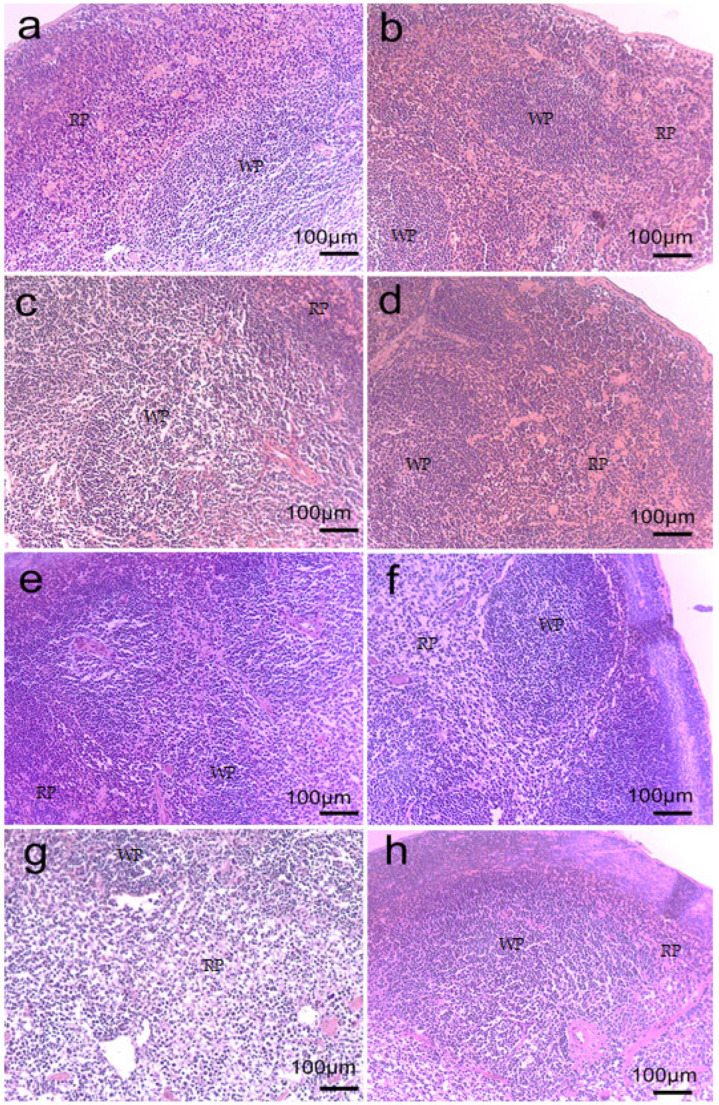
Morphology of the spleen of mice with CPh-induced immunosuppression after implantation of Fe–Mn–C+rG-CSF (**a**), Fe–Mn–C+FCS (**b**), Fe–Mn–Pd+rG-CSF (**c**), Fe–Mn–Pd+FCS (**d**) scaffolds or subcutaneous administration of rG-CSF (**e**) and FCS (**f**) solutions, in comparison with mice with CPh-induced immunosuppression without therapy (**g**) and intact control (**h**). WP, white pulp, RP, red pulp.

The spleen plays a significant role in the innate and adaptive immune responses. Thus, it is one of the most important organs of the immune system. The results after studying the spleen morphology of mice from different groups showed that the CPh course without subsequent therapy led to depletion of the cellular composition of the organ (Figure 7). This increases the risk of both anemia and immunosuppression, since the spleen normally holds a reserve of RBCs and lymphocytes. Thus, it is obvious that the CPh-induced immunosuppression is accompanied not only by neutropenia, but can also lead to pancytopenia.

In comparison with the CPh group, the treatment of mice by rG-CSF and FCS loaded into scaffolds or injected in solutions resulted in minimization of negative changes in the spleen morphology. This can be regarded either as a consequence of rapid restoration of the organ’s cellular composition or as a consequence of the protective effect of hemo-stimulating agents that prevented cellular depletion of the organ due to active stimulation of cell proliferation. Comparing the effect of the two hemo-stimulating agents, it should be noted that the use of rG-CSF contributed to the restoration of WBC concentrations in the spleen to a greater extent, whereas the effect of FCS resulted in equilibrium restoration of both WBC and RBC content in the organ tissue.

## 3. Discussion

The obtained results confirm previously published data on the hemo-stimulating activity of natural FCS extracted from the sea cucumber *C. japonica*, as well as its analogues extracted from other representatives of the sea cucumber, *Psolus peronii* and *Holothuria nobilis* [22,23]. According to this data, FCS solutions were injected subcutaneously 24 h after the end of the CPh course. This is a standard recommendation for the use of rG-CSF-based drugs for the reconstitution of hematopoiesis in patients after chemotherapy. In contrast, in the present study, hemo-stimulants, both loaded into scaffolds or used as solutions, were administered in mice immediately after the treatment with CPh. Nevertheless, the studies showed that FCS promotes balanced restoration of blood composition after the cytostatic course. Compared with rG-CSF, FCS had a similar safe effect on WBCs in the blood and spleen stroma. At the same time, the activity of FCS in stimulating the growth of RBCs in the spleen as well as neutrophils and platelets in the blood was slightly increased.

It is obvious that this result is determined by the partial similarity of the mechanism of therapeutic action of FCS and rG-CSF. The data obtained earlier show that both rG-CSF and sulfated polysaccharides, which include FCS extracted from sea cucumbers, inhibit the induction by cells of interleukin-6, which has an ambivalent effect on the immune system [22].

In comparison with subcutaneous injection of rG-CSF and FCS solutions, the implantation of a biodegradable scaffold loaded by active substances led to the prevention of pancytopenia. It resulted also in the restoration of platelet and hemoglobin concentrations in the mice blood, as well as maintaining the supply of RBCs and lymphocytes in the spleen. Since an increase in the concentration of Ki-67(+) cells was found in the bone marrow of mice with implanted scaffolds, it can be assumed that the delayed release of FCS and rG-CSF stimulated the proliferative potential of bone marrow hematopoietic cells, which was reduced after the CPh course. It is obvious that the mechanisms of stimulation of hematopoiesis by FCS and rG-CSF differ. As we have shown earlier, FSC stimulates the proliferation of stem hematopoietic cells (CD34+CD45+) and accordingly leads to the restoration of various links in hematopoiesis [22]. On the other hand, rG-CSF is the principal cytokine controlling neutrophil development and function [32].

This effect was achieved by reducing the bioavailability of hemo-stimulants that were immobilized in a scaffold covered with a polyethylene glycol film in the first hours after implantation. Polyethylene glycol was used to encapsulate the contents of the scaffold, preventing leakage and evaporation of Matrigel and protecting medical personnel from coming into contact with the hemo-stimulants during the process of implanting the device under the patient’s skin. The release of the active compound into the tissues surrounding the implant became possible only after the sequential biodegradation of the polyethylene glycol layer, the alloy of the scaffold wall and Matrigel, followed by diffusion of the hemo-stimulant into the surrounding tissues.

Therefore, the effect of the hemo-stimulant (rG-CSF or FCS) is realized after the removal of CPh from the body, which prevents the onset of severe immunosuppression. It can be assumed that rG-CSF and FCS, which entered from the implant, neutralized the negative effect of the cytostatic on blood cells due to increased proliferation of hematopoietic cells, contributing to the preservation of the cellular composition of the bone marrow and spleen.

It should be noted that the most pronounced effect of hematopoiesis stimulation under the influence of FCS was achieved after its introduction as part of a scaffold based on the Fe–Mn–C alloy after ECAP. It is possible that the advantage of the Fe–Mn–C alloy in this case was a lower rate of biodegradation compared to the Fe–Mn–Pd alloy after ECAP, which resulted in the maintenance of the kinetics of the hemo-stimulator entering the bloodstream that was optimal for hemo-stimulation. It is possible that it was the elemental composition of the scaffold that also contributed to a more significant manifestation of the planned therapeutic effect. Microelements released from the scaffold during its biodegradation can affect hematopoiesis through different mechanisms.

It is known that erythropoietic tissue is the main consumer of iron required for heme synthesis [33]. Iron plays a role in the proliferation and differentiation of hematopoietic cells by modulating the level of reactive oxygen species and activation of mitogen-activated protein kinase [34,35]. It has been shown that iron deficiency suppresses the differentiation of HL-60 promonocytes into macrophages/monocytes, enhancing apoptosis due to inhibition of cyclin genes, and also correlates with the level of lymphocyte proliferation [35,36,37]. Since manganese is actively involved in the synthesis and activation of many enzymes (oxidoreductases, transferases, hydrolases, isomerases and lyases), it is also involved in hematopoiesis [38]. In particular, Mn is a necessary cofactor for arginase, a superoxide dismutase that can suppress cellular oxidative stress and ensure normal maturation of erythrocytes [39]. The metabolism of Fe and Mn is closely related. These trace elements can not only alter the synthesis of erythrocytes, but can also affect the distribution and storage of blood cells in target organs, thereby changing the state of blood parameters in the body [40].

On the other hand, the reduced activity of Fe–Mn–Pd scaffolds compared to Fe–Mn–C may be explained by the negative effect of Pd ions on the proliferation of hematopoietic cells. It was previously shown that Pd ions are capable of prolonging the G1 phase of the cell cycle, thus preventing cell proliferation [41]. However, these considerations require additional verification. It should be noted that the main effect of prolonging the action of the studied substances is due not to the scaffold biocorrosion, but to the hydrogel in which rG-CSF or FCS is placed. The scaffold biocorrosion occurs slowly, at 0.63–0.97 mm per year and, as our previous studies have shown, it does not have cytotoxicity and does not cause pathological changes in the internal organs. There is much literature data on the effect of Fe/Mn/Pd/ions on the body, since they are components of medical devices implanted into the body for a long time, for example, dental casting alloys, which indicates the absence of any significant side effects of their biodegradation products [42].

In our previous work, we assessed the effect of implantation of an Fe–Mn-based alloy on changes in blood biochemistry parameters (total bilirubin, urea, creatinine and albumin in blood serum), correlating with impaired renal and hepatic function. No evidence of significant differences was found in comparison with intact animals [43].

A slightly different picture was observed after subcutaneous injection of FCS and rG-CSF solutions in mice immediately after the end of the CPh course. It can be assumed that, due to high bioavailability, FCS and rG-CSF began to realize a stimulating effect on cell growth in a short time even before the end of the cytopathogenic effect of the cytostatic. In this case, CPh canceled the effect of hemo-stimulants due to the direct cytotoxic effect on recruited cells in the systemic bloodstream, leading to their massive death. Potentially, such a scenario can provoke toxic damage to the liver and/or kidneys due to the increased load on these organs, due in turn to the need to remove a large volume of metabolites from the body.

In this case, the potential for therapy of CPh-induced immunosuppression, which developed as a result of pancytopenia, could be reduced due to the depletion of the number of blood cells deposited in the bone marrow and spleen.

The achieved results may be of interest for the development of a device that could be implanted in a cancer patient simultaneously with the injection of a chemotherapy drug in order to prevent complications such as pancytopenia, which can occur due to the delayed onset of the hemo-stimulating effect of the active substance. The use of biodegradable components eliminates the need for surgical intervention to remove the implant parts after the end of its therapeutic effect. The potential therapeutic benefits of this design should be further explored through a series of subsequent experiments. This approach aims to reduce hospital visits for patients on the first day after chemotherapy, a period that is usually the most difficult for patients to tolerate, and to reduce the risk of a sudden drop in blood cell concentration due to the programmed timely start of maintenance therapy.

## 4. Materials and Methods

### 4.1. Sulfated Polysaccharides

The FCS sample was extracted from a body wall of the sea cucumber *C. japonica*, as described previously [44]. FCS was then dissolved in Hanks’ solution (0.5 mg/mL) and sterilized by perfusion through a 0.22 μm filter (Corning, Glendale, CA, USA).

### 4.2. Scaffolds

The scaffold shells were made of the Fe–Mn-C and Fe–Mn-Pd alloys melted in a Leybold Heraeus (VAR) L200DI vacuum arc remelting furnace (Leybold, Cologne, Germany) from technically pure iron with the addition of electrolytic manganese (≈99.8% Mn). The chemical composition of the alloys is presented in Table 2.

**Figure 8:**
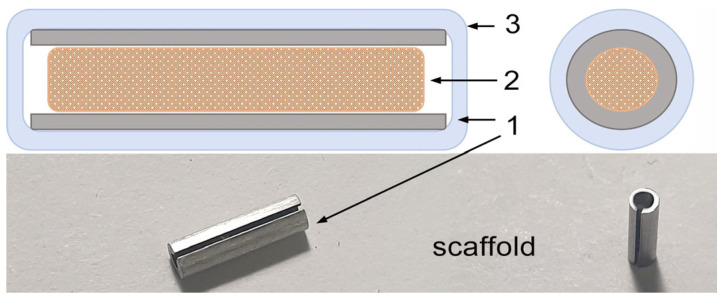
Scaffold scheme: 1—hollow cylinder made of Fe–Mn–C and Fe–Mn–Pd alloys; 2—mixture of gel and the preparation; 3—outer layer (polyethylene glycol).

The alloys were subjected to homogenizing annealing followed by ECAP. To maximize structure refinement, the Fe–Mn–C alloy samples were processed with six ECAP passes and the Fe–Mn–Pd alloy samples at 450 °C were processed with four ECAP passes. The obtained samples were used to manufacture tubular scaffold shells with a length of 8 mm and an internal diameter of 2 mm (Figure 8).

The scaffold shells were washed in distilled water in an ultrasonic washer and then kept in 70% ethanol for 4 h. After drying, the shells of the Fe–Mn-C and Fe–Mn-Pd alloy scaffolds were filled with Matrigel (Corning, USA) containing 0.1 mg of FCS solution (Fe–Mn-C+FCS and Fe–Mn-Pd+FCS, respectively) or 0.6 nmol rG-CSF (Leucita, Sygardis AqVida, Germany) (Fe–Mn-C+rG-CSF and Fe–Mn-Pd+rG-CSF, respectively), kept at 37 °C for gel polymerization, immersed in sterile 6% polyethylene glycol solution and incubated at 37 °C until the start of the experiment. The loaded scaffolds were used no later than 4 h after preparation.

### 4.3. Animal Model

The animal protocols used in this work were evaluated and approved by the local ethical committee of the N.N. Blokhin National Medical Research Center of Oncology (Protocol 12-2024). They are in accordance with the order 490 (5 November 2008) of the Agricultural Ministry of Russian Federation and meet National GLP Standard of Russian Federation [45] and European Convention for the Protection of Vertebrate Ani-mals used for Experimental and Other Scientific Purposes (Strasbourg, France, 18 March 1986).

Forty-eight mice of the Balb/c line (males, weight 21 ± 2 g) were divided into 8 groups with 6 animals in each group. Before and during the experiment, the animals were in standardized vivarium conditions (at 20 ± 2 °C with free access to food and water). For the inducing of myelosuppression, CPh (Endoxan, Baxter, Germany) at a dosage of 100 mg/kg was injected to animals of 7 groups once daily parenterally for 3 days.

Intact animals were designated as the Intact Control group. Immediately after the last injection, animals were either implanted subcutaneously with prepared scaffolds (Fe–Mn-C+FCS, Fe–Mn-Pd+FCS, Fe–Mn-C+rG-CSF, and Fe–Mn-Pd+rG-CSF groups) or injected subcutaneously with 0.2 mL of FCS or 0.6 nmol rG-CSF. One group of animals with CPh-induced immunosuppression was left without treatment (CPh). The animals were euthanized after 3 days.

Blood and bone marrow were stabilized by ethylenediaminetetraacetic acid (EDTA), spleen was fixed by 10% formalin and stained with hematoxylin-eosin (HE). In the blood, we studied the concentration of white blood cells (WBCs) and, in particular, neutrophils (Neu), lymphocytes (Lym), monocytes (Mono) and eosinophils (Eos), as well as the concentration of red blood cells (RBCs), hemoglobin (HGB), and platelets (PLT). These hematologic parameters were analyzed with an automatic analyzer Diatron Abacus Junior 30 (Diatron, Budapest, Hungary).

The proliferative activity of bone marrow cells was assessed by determining the con-centration of cells labeled with the Ki-67 (Ki-67(+) cells) using the Muse Cell Analyzer (Millipore, Darmstadt, Germany) and the Muse^®^ Ki67 Proliferation Kit (Thermo Fisher Scientific, Waltham, MA, USA) in accordance with the manufacturer’s instructions.

### 4.4. Statistical Analysis

The results of the individual parameter evaluation were presented in the form of the mean (M) and standard deviation (SD). To evaluate the hematological parameters, the studies were performed in triplets. To evaluate the proliferative activity, at least 5000 cells were examined in each sample. A comparative analysis was performed using the median test. Differences were considered significant at *p* < 0.05.

## 5. Conclusions

In this study, a biodegradable scaffold loaded with FCS isolated from the sea cucumber *C. japonica* or rG-CSF was implanted into mice. The result of such use of the scaffold was a gradual release of the hemo-stimulating agent into the systemic bloodstream after the elimination of the cytostatic agent from the body. A further investigation demonstrated a decrease in the severity of pancytopenia after the completion of cyclophosphamide treatment. This effect was manifested in a significant increase in the number of WBCs and neutrophils, in particular platelets, in the blood of animals, as well as a decrease in the severity of anemia in animals with implants compared to the control group without treatment. This result was more pronounced than with subcutaneous administration of FCS or rG-CSF.

This effect of the implants developed, together with an increase in the concentration of Ki-67(+) cells in the bone marrow, indicates the stimulation of their proliferation, as well as elevation of the content of lymphocytes and RBCs in the spleen, which is one of the main organs of the immune system.

It was found that the most significant effect of hemo-stimulation occurred in animals after the implantation of scaffolds made from the Fe-27Mn-0.2C after ECAP. Compared to Fe-22Mn-0.9Pd, this alloy has a lower biodegradation rate (this was confirmed by in vitro experiments), which allowed the active compounds to enter the blood after CPh had been eliminated from the body. In addition, the elements released during the alloy biodegradation (primarily iron) were also potentially capable of providing an additional hemo-stimulating effect. The developed design appears to be a promising model of a medical device for reducing the severity of pancytopenia in cancer patients undergoing chemotherapy due to the preventive administration of hemo = stimulators with delayed effect. The use of FCS from sea cucumber *C. japonica* as an active agent will expand the arsenal of hemo-stimulants, reducing the risk of developing immunosuppression and thrombocytopenia.

The comparison of hemo-stimulating activities of FCS and rG-CFS was previously studied by us in experiments on cyclophosphamide-induced mice. This demonstrated the advantage of FCS over rG-CFS because of the ability of FCS to stimulate not only neutropoiesis but all links of hemopoiesis [19,20]. Herewith, we reported the formulation of FCS in the implantable bioresorbable scaffold to manage a prolonged action of FCS and a delayed stimulation of hematopoiesis. Thus, the formulation of FCS into the scaffold permitted the achievement of these goals and improved the character of the pharmacological effect of FCS. It should be noted that further veterinary and clinical studies towards the practical application of developed FCS-formulation need deeper investigation of the kinetics of implant resorption and possible associated undesirable biological actions. Such study is in progress; its results will be reported by us in due course.

## Figures and Tables

**Table 1:** Properties of the Fe–Mn alloys and Fe after annealing and ECAP.

№	Alloys	Treatment	σ_UTS_ [MPa] ^1^	σ_YS_ [MPa] ^2^	ε [%] ^3^	C_R_ [mm/y] ^4^	Ref.
1	Fe–Mn–C	ECAP at 450 °C (N = 6)	1515	1327	12.5	0.63	[30]
2	Fe–Mn–Pd	ECAP at 450 °C (N = 4)	1669	1577	4	0.97	[29]

^1^ σ_UTS_—ultimate tensile strength [MPa]; ^2^ σ_YS_—yield strength [MPa]; ^3^ ε—total elongation [%]; ^4^ C_R_—corrosion rate [mm/y].

**Table 2:** Chemical composition (in wt.%) of the alloys.

Alloy	Mn	Pd	Si	Cu	C	P	S	Fe
Fe–Mn-C	26.9	-	0.496	0.576	0.213	0.015	0.007	Bal.
Fe–Mn-Pd	22.20	0.88	0.78	0.38	0.01	0.013	0.005	Bal.

## Data Availability

All the data required to reproduce these experiments are present in the article. Raw materials can be provided upon special request.

## Abbreviations

The following abbreviations are used in this manuscript:

rG-CSF	Recombinant human granulocyte colony stimulating factor, filgrastim
sTNF-Rs	Soluble tumor necrosis factor receptors
GM-CSF	Granulocyte-macrophage colony-stimulating factor, lenograstim
WBCs	White blood cells
RBCs	Red blood cells
HGB	Hemoglobin
PLT	Platelets
Neu	Neutrophils
Lym	Lymphocytes
Mono	Monocytes
Eos	Eosinophils
FCS	Fucosylated chondroitin sulfate
CPh	Cyclophosphamide
ECAP	Equal channel angular pressing
EDTA	Ethylenediaminetetraacetic acid
HE	hematoxylin-eosin
